# Rational mutagenesis by engineering disulphide bonds improves *Kluyveromyces lactis* beta-galactosidase for high-temperature industrial applications

**DOI:** 10.1038/srep45535

**Published:** 2017-03-31

**Authors:** Agustín Rico-Díaz, María-Efigenia Álvarez-Cao, Juan-José Escuder-Rodríguez, María-Isabel González-Siso, M. Esperanza Cerdán, Manuel Becerra

**Affiliations:** 1 Universidade da Coruña. Grupo EXPRELA, Centro de Investigacións Científicas Avanzadas (CICA), Departamento de Bioloxía Celular e Molecular, Facultade de Ciencias, A Coruña, Spain

## Abstract

*Kluyveromyces lactis* β-galactosidase (Kl-*β*-Gal) is one of the most important enzymes in the dairy industry. The poor stability of this enzyme limits its use in the synthesis of galactooligosaccharides (GOS) and other applications requiring high operational temperature. To obtain thermoresistant variants, a rational mutagenesis strategy by introducing disulphide bonds in the interface between the enzyme subunits was used. Two improved mutants, R116C/T270C and R116C/T270C/G818C, had increased half-lives at 45 °C compared to Kl-*β*-Gal (2.2 and 6.8 fold increases, respectively). Likewise, Tm values of R116C/T270C and R116C/T270C/G818C were 2.4 and 8.5 °C, respectively, higher than Kl-*β*-Gal Tm. Enrichment in enzymatically active oligomeric forms in these mutant variants also increased their catalytic efficiency, due to the reinforcement of the interface contacts. In this way, using an artificial substrate (*p*-nitrophenyl-β-D-galactopyranoside), the Vmax values of the mutants were ~1.4 (R116C/T270C) and 2 (R116C/T270C/G818C) fold higher than that of native Kl-*β*-Gal. Using the natural substrate (lactose) the Vmax for R116C/T270C/G818C almost doubled the Vmax for Kl-*β*-Gal. Validation of these mutant variants of the enzyme for their use in applications that depend on prolonged incubations at high temperatures was achieved at the laboratory scale by monitoring their catalytic activity in GOS synthesis.

β-D-galactosidases (EC 3.2.1.23) are enzymes that catalyse the hydrolysis of terminal non-reducing β-D-galactose units from β-D-galactosides. They have been mainly used for the hydrolysis of lactose in milk and other dairy products. β-galactosidases also have transgalactosylation activity that make them very attractive for obtaining galactooligosaccharides (GOS)^1^. GOS are prebiotic milk derivatives, which nowadays are included in some foods^2^ and that have been suggested to be beneficial to consumer’s health, such as prevention of colorectal cancer^3^, avoidance and treatment of symptoms in asthmatic disease^4^, or improvement in the microbiota and certain markers of immune function in elderly people^5^. The convenience of adding GOS to infant formula has also been proposed recently, since there seems to be some beneficial effects on gut microbiota, metabolic activity, stool consistency and frequency, and the amelioration of certain immune markers in babies^6^. The biggest drawback of the enzymatic production of GOS comes from the need for working at high lactose concentration, since transgalactosylation is favoured under these conditions over other enzymatic activities. The poor solubility in water of this disaccharide means that transgalactosylation reactions must run at relatively high temperatures to reach the required lactose concentration in solution^7^.

*β*-D-Galactosidase from the yeast *Kluyveromyces lactis* (Kl-*β*-Gal) is widely used in the food industry. However, the stability of native Kl-*β*-Gal^8^ limits its catalytic activity in applications that need high temperatures, e.g. the production of GOS^9^. Even though thermophilic bacteria might offer more versatility for obtaining robust enzymes for this technology, the confirmed GRAS (Generally Recognized As Safe) condition of yeasts, like *K. lactis* and *K. marxianus*, and fungi, like *Aspergillus niger* and *A. oryzae*, places them among the favourite sources^10^. Some approaches have been performed with Kl-*β*-Gal to improve its thermal stability; these include immobilization using functionalized multi-walled carbon nanotubes^11^, quitosan particles^12^ or polystyrene nanofibers^13^.

Protein engineering is widely used to increase enzyme stability^14^ by using directed evolution methods (error prone PCR and DNA shuffling), semi-rational methods such as CASTing, or rational methods supported by the study of the structure of the enzymes^15^,^16^,^17^. However, there are currently no published reports on protein engineering strategies for improving the thermal stability of Kl-*β*-Gal.

From our structural studies^18^, we found that Kl-*β*-Gal (PDB code 3OBA) is a tetrameric enzyme with an oligomerization pattern of “dimerization of dimers”, with a higher dissociation energy for the dimers than for the tetramer. Based on this knowledge and in depth analysis of its catalytic sites^18^, we hypothesized that design strategies used in other systems^19^ based in the substitution of some target residues in the contact interfaces between dimers, thus enabling new stabilizing interactions^19^, could effectively increase the thermal stability of the enzyme.

We present here work based on this rational strategy for improvement of the thermoresistance of the Kl-*β*-Gal enzyme. The catalytic and stability properties of 2 active variants obtained by introducing disulphide bonds between the oligomers are compared with the native enzyme. Their potential value in industrial applications needing high temperatures has been validated by measuring their transgalactosylation activity in GOS production.

## Results and Discussion

### Design of a stabilization strategy for Kl-*β*-Gal

**Figure 1:**
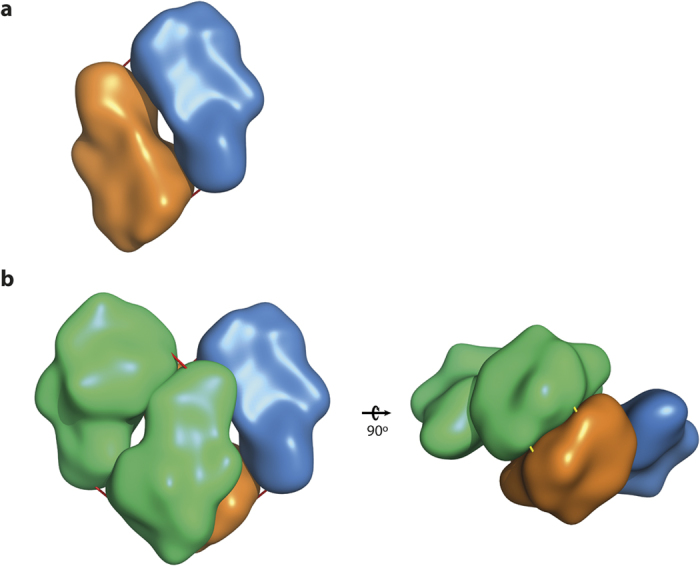
Surface representation of Kl-*β*-Gal topology showing the oligomeric organization. (**a**) Dimer made up by subunits A (orange) and C (blue). Red bars represent the location of disulphide bonds between Cys3 (native) and Cys818 in mutant R116C/T270C/G818C. (**b**) Tetramer made up by dimer of subunits B and D (green), and dimer of subunits A (orange) and C (blue). Red bars represent the location of disulphide bonds between Cys3 (native) and Cys818 in mutant R116C/T270C/G818C and yellow bars represent the location of disulphide bonds between Cys116 and Cys270 in mutants R116C/T270C and R116C/T270C/G818C.

Kl-*β*-Gal adopts 2 active oligomeric forms in solution, as a dimer or a tetramer formed by a dimer of dimers^18^. The predicted dissociation energy of the dimers in the tetramer is lower than that of the monomers in the dimer. The structure of the combined monomers (4 identical subunits A, B, C, D) in the tetramer and dimers is shown in Fig. 1. Although not a general rule, there is evidence supporting the notion that, in families of multimeric enzymes, members with the highest thermal stabilities also have the highest oligomerization states^20^,^21^,^22^. Reduction in the accessible surface area produced by the joining of the subunits has also been associated with thermostability^23^. As a result of this rational strategy, different proteins (e.g. malate dehydrogenase and cocaine esterase) have been successfully engineered to form and stabilize oligomers^24^,^25^.

Kl-*β*-Gal has detectable enzymatic activity only in its dimeric and tetrameric forms^26^, which can be attributed to modifications at the entrance to the catalytic pocket after dimerization of the monomers^18^. Therefore, our stabilization strategy has been focused on the reinforcement of Kl-*β*-Gal quaternary structure. To strengthen the interface contacts between monomers and between both dimers, a prediction of putative new inter-molecular interactions was made by investigating the interface areas of the oligomer using 2 molecular visualization softwares, Pymol and Coot.

Along with these considerations derived from studies of oligomer interface surfaces using several molecular graphic softwares described in Methods, the selection of target residues also took into account features such as B-factor values and stability changes produced by the newly introduced amino acid residues. Residues with the highest average B-factors correspond to those with the highest thermal motion and flexibility of a protein^27^. Inversely, there is a direct relationship between the degree of rigidity and protein thermostability^23^. The properties of the modified molecular interactions due to these substitutions, the residue distances, dihedral angles and energetic constrains were also calculated.

Three mutation targets (pairs Val2-His817, Gly37-Ser260 and Gly818-Cys3) were selected from the monomer-monomer contact surfaces of the dimers (between subunits A-B or C-D, Fig. 1b) and 4 (pairs Asp116-Thr270, His425-Tyr872, Leu601-Glu922 and Gly983-Gly983) from the main dimer-dimer interface (between subunits A and B, Fig. 1a; Table 1). In all cases, the selected residues were mutated to cysteines to get new disulphide bonds due to the covalent nature of these molecular interactions, which could maximize reinforcement of the interface surfaces.

Therefore, to insert disulphide bonds at the monomer-monomer interface, mutants V2C/H817C, G37C/S260C and G818C were obtained. However, the mutants R116/T270, H425C/T872C, L601C/E922C and G983C were generated to form disulphide bridges at the dimer-dimer interface.

### Screening and verification of mutants

Protein extracts from cells carrying these variants were analysed by measuring thermal stability and residual catalytic activity, using β-PNPG as the substrate. Thermal stabilities were tested after 20 min incubation at 42.5 °C and compared to the residual activity of the native enzyme given the same treatment. In 3 cases (G37C/S260C, G818C and G983C), there was no increase in their residual activity compared with the native enzyme. Furthermore, the residual activity of 2 mutants (V2C/H817C and L601C/E922C) was 10% lower, and H425C/T872C had no enzymatic activity (Table 1). This undesirable feature was interpreted as a consequence of the breakage of other important intermolecular linkages between the subunits, e.g. hydrogen bonds.

**Figure 2:**
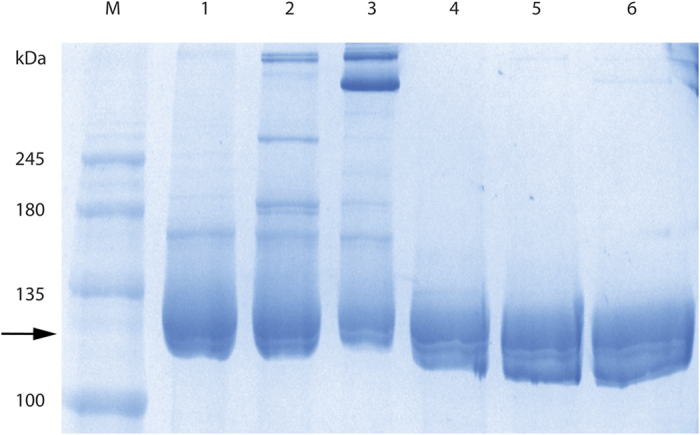
SDS PAGE of Kl-*β*-Gal and its mutants in oxidizing and reducing conditions. M, protein MW marker; 1, Kl-*β*-Gal without 2-mercaptoehtanol; 2, R116C/T270C without 2-mercaptoehtanol; 3, R116C/T270C/G818C without 2-mercaptoehtanol; 4, Kl-*β*-Gal with 2-mercaptoehtanol; 5, R116C/T270C with 2-mercaptoehtanol; 6, R116C/T270C/G818C with 2-mercaptoehtanol. The arrow points to the monomer form (118 KDa) and upper forms represent diverse high-order forms. The quantity of the monomer form diminishes gradually in 1, 2 and 3, whereas in 4, 5 and 6 it remains constant.

Interestingly, R116C/T270C which has residue changes located in the dimer-dimer interface, had a noticeable increase (>10%) in the residual activity (Table 1). Analysis of this variant by SDS PAGE, with and without addition of β-mercaptoethanol used to break disulphide bonds, showed the presence of larger complexes of this mutant enzyme under oxidizing conditions, thus suggesting the existence of disulphide bonds between both dimers (Fig. 2). Because a pair of mutated oligomers contributes to the dimer-dimer interface, 2 disulphide bonds are formed that further strengthen the final structure of the tetramer (Fig. 1b).

An explanation for the success of this variant requires several factors to be considered simultaneously. In addition to the optimal bond distance between Asp116 and Thr270 in the different conformations analysed (1.80–2.25 Å), the relatively high average B-factors of these residues (34.70 and 40.98 compared with the B-factor molecule average of 19.82) make them very flexible and suitable for stabilization purposes. Therefore, the reduction in the thermal motion of these two flexible amino acids by the introduction of a new covalent bond in our design has made the area more rigid and the tetramer more stable. On the other hand, threonine is chemically analogous to cysteine; thus substitution in the position 270 does not affect other molecular contacts^24^,^28^, and hence the effects of unfavourable protein unfolding have been avoided in this way.

After obtaining the R116C/T270C mutant, which improved the thermoresistance of Kl-*β*-Gal by stabilizing the contacts between dimers, our rational strategy was used to reinforce the other subunit interfaces (monomer-monomer). We mutated R116C/T270C by introducing a cysteine in the 818 position. The single mutant, G818C, was the only mutation in the monomer-monomer interface that did not lower thermotolerance and catalytic activity (Table 1), which was the reason testing its possible synergistic effect of the accumulated changes in the triple mutant.

Table 1 shows that R116C/T270C/G818C retains more enzymatic activity (25% more than the native enzyme) after heat treatment than R116C/T270C (only 10% higher). This suggests an accumulative effect of the 2 mutations. SDS-PAGE analysis of purified R116C/T270C/G818C under oxidizing and reducing conditions also indicates differences from R116C/T270C. The bands corresponding to tetramer forms increase under oxidizing conditions, probably due to the formation of additional disulphide bonds on R116C/T270C/G818C (Fig. 2). The new bonds in the mutant are expected to be 4 in the tetramer because of the oligomerization features of Kl-*β*-Gal (Fig. 1).

### Verification of disulphide bond formation

To confirm the disulphide bonds formation, two approaches were done: a mass spectrometry and a colorimetric assay.

By MS/MS analysis mass modifications in cysteines of the mutant variants involved in theoretical inter-chain disulphide bonds were detected. These mass modifications are compatible with the previous existence of a disulphide bridge that was asymmetrically broken by fragmentation, resulting on the presence of dehydroalanine (dhA) in one of the two sites involved in the contact^29^,^30^.

In this way, both in R116C/T270C and in R116C/T270C/G818C mutants, dhA modifications in residue Cys116 were detected. Moreover, analyses showed dhA modifications in residue Cys270 of R116C/T270C/G818C variant. Thus, these data support the formation of disulphide bonds between the two cysteines introduced in the dimer-dimer interface of the enzyme.

On the other hand, in mutant R116C/T270C/G818C dhA on Cys3 site was detected, which makes a disulphide contact with Cys818 according our theoretical model. Unfortunately, peptide fragment that contains Cys818 is highly hydrophilic, which makes difficult its detection (Table 2).

Additionally, the formation of disulphide contacts in Kl-*β*-Gal and its mutants was also examined using DTNB [5,5′-dithiobis (2-nitrobenzoic acid)], which reacts with free sulfhydryl groups of the protein structure. The differences between the results in non-reduced and reduced conditions were significant in both mutants, R116C/T270C and R116C/T270C/G818C, while there were no significant changes between conditions in native Kl-*β*-Gal. Moreover, differences were more pronounced in variant R116C/T270C/G818C. These data show that R116C/T270C/G818C has a larger proportion of cysteines joined as disulphide bonds than R116C/T270C variant, as it was predicted (Table 3).

All of these results together and the improvement observed in residual activity support the presence of new disulphide contacts in the mutant enzymes obtained. The number of new disulphide bonds is higher in R116C/T270C/G818C mutant because of the additionally effect of mutation G818C, which confers new contacts in monomer-monomer interface, and in turn amplifies the residual activity improvement.

### Stability analysis of the two selected mutants

**Figure 3:**
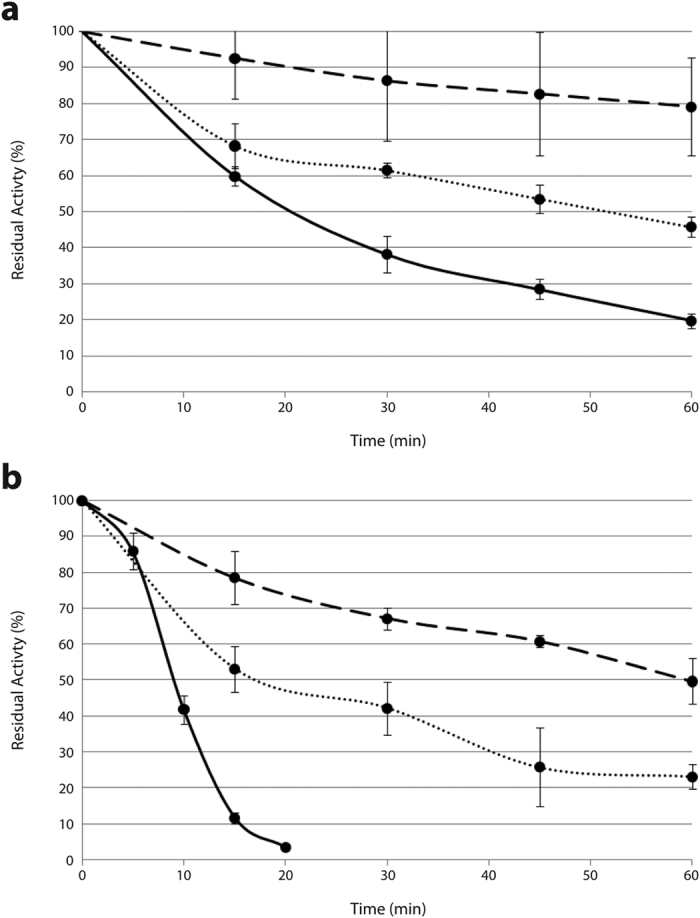
Effect of temperature on residual activity of each Kl-*β*-Gal variant. (**a**) 40 °C treatment; (**b**), 45 °C treatment. Solid lines represent native Kl-*β*-Gal, dotted lines represent R116C/T270C, and dashed lines represent R116C/T270C/G818C. Error bars are standard deviations of 3 measurements.

Analyses carried with highly purified protein samples, native Kl-*β*-Gal and the 2 selected mutants (R116C/T270C and R116C/T270C/G818C), confirmed the improved stability of the variants found during screening. Stability was tested by incubating samples at 40 and 45 °C. After incubating R116C/T270C for an hour at 40 °C, the mutant enzyme retained 45% activity, whereas the native enzyme only retained 20% of its activity (Fig. 3a). After 1 h at 45 °C, this mutant retained 25% of the β-galactosidase activity, but the native enzyme was almost completely (96–97%) inactivated within 20 min (Fig. 3b). After 1 h at 40 °C or 45 °C in R116C/T270C/G818C, the enzyme retained 79% and 49% of its initial catalytic activity, respectively (Fig. 3a and b).

In terms of half-life at 45 °C, Kl-*β*-Gal’s was 26.11 min, whereas R116C/T270C and R116C/T270C/G818C half-lives were 57.64 and 178.51 min, respectively. This shows an important increase in the mutant, 2.2 fold for R116C/T270C and 6.84 fold for R116C/T270C/G818C, with reference to the native enzyme’s half-life.

**Figure 4:**
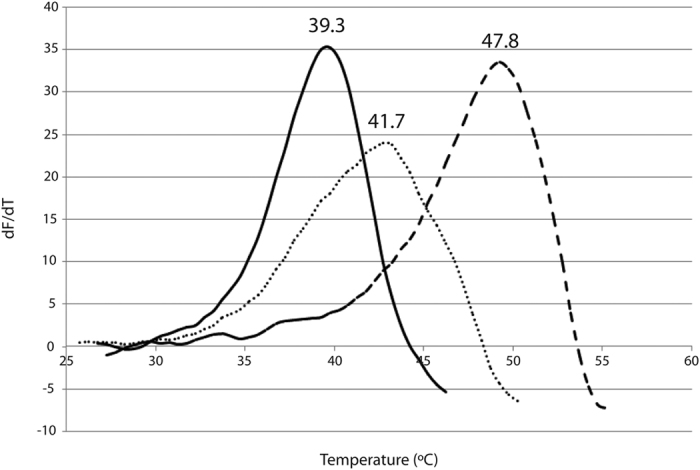
DSF analysis. dF/dT is plotted against temperature. The maximum of the fitted curve is the melting point (Tm) of the protein. Solid lines represent native Kl-*β*-Gal, dotted lines represent R116C/T270C, and dashed lines represent R116C/T270C/G818C.

DSF assay of the 3 enzyme variants was used to measure and compare their Tm values. R116C/T270C (Tm = 41.7 °C) and R116C/T270C/G818C (Tm = 47.8 °C) have 2.4 °C and 8.5 °C higher values than the Tm of the native enzyme (39.3 °C) (Fig. 4).

These data show a significant improvement in thermostability achieved in R116C/T270C/G818C in reference to R116C/T270C, which in turn had higher thermal resistance than native Kl-*β*-Gal.

### Kinetic analysis of hydrolytic activity in the two selected mutants

Kinetic characterization of the 2 selected variants with the artificial substrate *p-*nitrophenyl-β-D-galactopyranoside (β-PNPG) in comparison with the native form showed kinetic differences (Table 4). The 3 forms had similar affinities for this substrate, as deduced from their Km values with values between 2.15 and 2.47. However, the mutant enzymes both had a higher Vmax (99.42 U/mg for R116C/T270C and 146.3 U/mg for R116C/T270C/G818C) than native Kl-*β*-Gal (71.9 U/mg). Considering that the same protein concentration had been used in all cases, the results clearly show that R116C/T270C and R116C/T270C/G818C have higher catalytic efficiency than the native protein. The differences are more pronounced in R116C/T270C/G818C, doubling values (146.3 U/mg *versus* 71.9 U/mg) found with the native enzyme (Table 4).

By kinetic analysis with lactose, a natural substrate, the affinities for the three enzymes were also similar (Km values between 34.31 and 38.20). Unexpectedly, values of Vmax of Kl-*β*-Gal and R116C/T270C were similar (15.99 U/mg and 15.22 U/mg), in contrast with values found with the β-PNPG substrate. However, the triple mutant, R116C/T270C/G818C, was significantly different from the other 2 enzyme forms in almost doubling their Vmax (28.48 U/mg) (Table 4).

Improvement in the Vmax of the catalytic hydrolysis by protein engineering strategy, especially remarkable in the case of R116C/T270C/G818C, has industrial applications. Processes, such as milk lactose hydrolysis or the development of whey syrups, used in human alimentation and pharmaceutical intermediates^1^,^10^,^31^, could benefit greatly by using this newly modified enzyme. A higher efficiency in the hydrolysis of substrates would yield the same amount of products with smaller amounts of protein, thereby reducing production costs.

### Oligomerization pattern analysis

**Figure 5:**
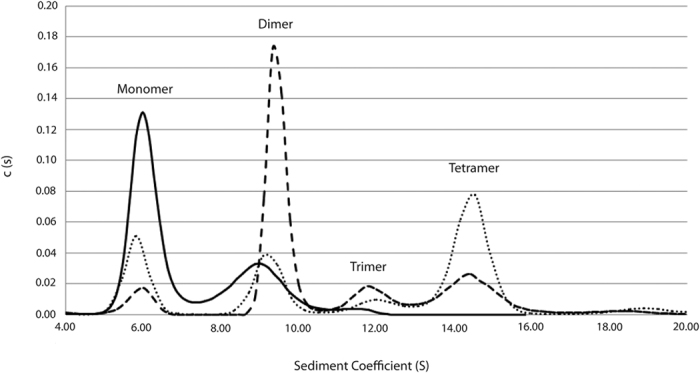
Analytical ultracentrifugation sedimentation velocity profile of the 3 enzyme variants (0.2 mg/mL). Kl-*β*-Gal (solid line), R116C/T270C (dotted line), and R116C/T270C/G818C (dashed line). Each peak is compatible with the theoretical sizes of different structural organizations: monomer, dimer, trimer and tetramer.

Analytical ultracentrifugation of variants under native conditions and at the highest enzyme concentration tested (0.2 mg/mL) showed differences in the oligomerization pattern (Fig. 5). As before^18^, the assay could only detect one of the active forms for the native enzyme, - the dimeric one - which represents 33.1% of the total. However, most of the protein was monomeric (66.9%).

R116C/T270C under the same conditions, however, had a more diverse oligomerization profile of molecular forms: monomer (21.9%), dimer (20.9%), trimer (7.9%) and tetramer (49.3%).

Finally, R116C/T270C/G818C had an oligomerization profile made up mainly of complex molecular forms, such as dimer (57.2%), trimer (12.6%) and tetramer (23%), with the monomer only representing 7.3% of the total.

Using a low protein concentration (0.1 mg/mL), the results with the mutants were comparable to those obtained at a higher protein concentration, but with a differential increase in monomeric forms of 5.5 (Kl-*β*-Gal), 0.8 (R116C/T270C) and 2.1% (R116C/T270C/G818C), respectively, and, not unexpectedly, a concomitant lowering of enzymatic activity occurred. This suggests protein concentration and high order oligomeric species are positively correlated in the native Kl-*β*-Gal and its mutants. The biggest increase corresponds to the native enzyme, probably because the initial equilibrium between oligomeric forms is displaced more towards monomers, thereby making it easier to observe this effect. Similar changes in oligomeric distribution at different protein concentrations have been observed for other proteins^32^,^33^,^34^.

**Figure 6:**
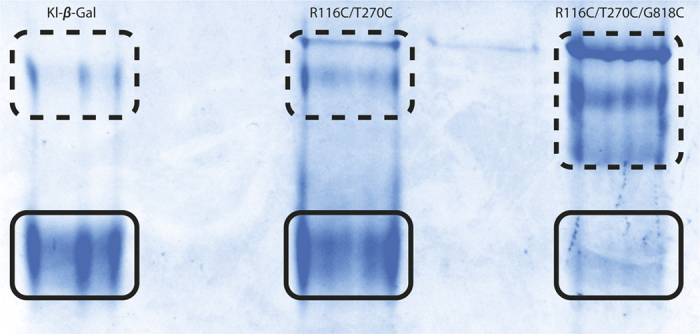
Native PAGE of Kl-*β*-Gal and its mutants. Solid line squares surround monomers and dashed line squares show oligomeric forms of each variant.

Polyacrylamide Gel Electrophoresis (PAGE) in native conditions was also used to confirm these variations in the oligomer distribution (Fig. 6), similar patterns being obtained as those in analytical ultracentrifugation experiments. While native enzyme is organized primarily in monomers, but showing also some oligomeric forms, both variants had considerably more oligomers. This is particularly clear in the analysis of R116C/T270C/G818C in showing a very low proportion of monomers.

These results support the higher propensity of the variants to form stable oligomeric species than the native enzyme, thus explaining their increased enzymatic activity and stability. The increase in the proportion of active forms in R116C/T270C and R116C/T270C/G818C could explain the improved efficiency of hydrolysis by the engineered enzymes, as seen in the kinetic analysis with β-PNPG as the substrate. Likewise, the higher proportion of oligomeric forms in R116C/T270C/G818C compared with R116C/T270C explains the increase of Vmax of the triple mutant with both substrates.

The slight differences in the improvement of Vmax between Kl-*β*-Gal and R116C/T270C in kinetic analyses with lactose or β-PNPG as substrates (Table 4) may be due to the relatively high protein concentration required for this method. As explained above, high protein concentration favours displacement of the equilibrium between the oligomeric forms towards higher order forms.

In conclusion, both mutants, but principally R116C/T270C/G818C, showed under all conditions tested an important improvement in activity compared with the native enzyme.

### Advantages of enzyme variants for galactooligosaccharide production

**Figure 7:**
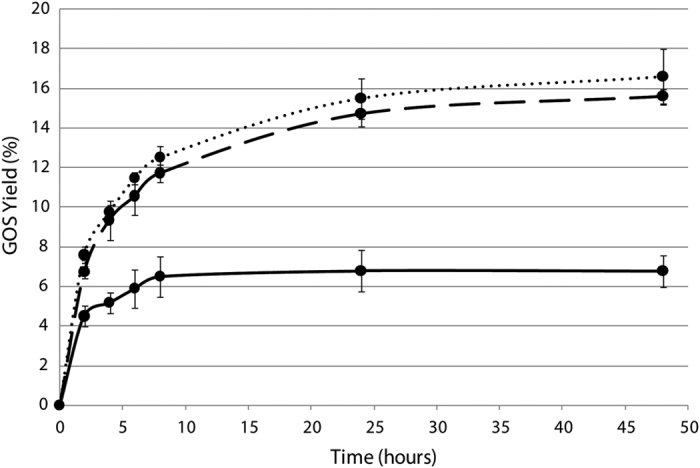
GOS (trisaccharides + tetrasaccharides) yield of different Kl-*β*-Gal variants in the synthesis assay with time. Solid lines represent native Kl-*β*-Gal, dotted lines represent R116C/T270C, and dashed lines represent R116C/T270C/G818C. Error bars are standards deviation of 3 measurements.

Measurements of transglycosylation activity along with increased thermotolerance described above show that maximum GOS yields are higher with R116C/T270C and R116C/T270C/G818C than the native enzyme (Fig. 7). Although both variants yield similar GOS production, it is noteworthy that there are the same number of enzymatic units in the reaction mixture of each of them. Therefore, taking into account the best kinetic efficiency of R116C/T270C/G818C, this triple mutant is more suitable for this application since same GOS production is obtained with less enzyme. In fact, R116C/T270C/G818C shows more specific productivity (g GOS·mg^−1^·h^−1^) at 2 h than R116C/T270C, 0.31 and 0.22 respectively.

These results support the suitability of these variants for applications that involve longer incubation times of the enzyme at relatively high temperatures. For example, new strategies could be designed in the production of GOS, by using less enzyme, which represents an important saving in the processing costs. R116C/T270C/G818C would also allow the use of higher temperatures for the assay; since enzyme denaturation is reduced, higher concentrations of lactose can be used, and consequently higher maximum yields of GOS can be achieved. The scaling-up of production and its optimization using these engineered enzymes can have significant industrial benefits.

## Conclusions

We have engineered 2 Kl-*β*-Gal variants by rational mutagenesis based on the structure of the enzyme, introducing disulphide bonds in monomer-monomer and dimer-dimer interfaces. The 2 mutants, R116C/T270C and R116C/T270C/G818C, had improved thermostability as measured by residual activity after incubation at 45 °C, and also increased half-lives and Tm values compared to native enzyme under the same conditions. Kinetic parameters corresponding to the hydrolytic reaction confirmed the improvement of the catalytic activity of the 2 mutant enzymes with the artificial substrate (β-PNPG), and in the case of R116C/T270C/G818C with the natural substrate, lactose. These improvements do not affect the affinity for the substrates, but rather is a consequence of an increase in Vmax. It has been experimentally confirmed that in both mutants, but especially R116C/T270C/G818C, both these improvements correlate positively with an increase in the proportion of dimeric and tetrameric species, the active forms of Kl-*β*-Gal^26^.

The applicability of both mutant variants in high temperature industrial applications and interest of their use have been validated by GOS synthesis assays.

## Methods

### Gene cloning and protein purification

The *LAC4* gene (Gene ID 2897170) was amplified by PCR from the pLX8 plasmid and cloned in the YEpFLAG vector (*Eastman Kodak Company*) as previously reported^35^. Mutagenesis of Kl-*β*-Gal was done by PCR with the commercial kit Quikchange-XL (*Stratagene*), oligonucleotide design and mutagenesis following the manufacturer’s recommendations. The construction was used to transform *Saccharomyces cerevisiae* BJ3505 cells (*Eastman Kodak Company*) with the commercial kit *Frozen-EZ Yeast Transformation Kit II™ (Zymo Research)*.

For protein extraction and purification, cells were grown at 30 °C and 250 rpm for 72 h in a 2 L Erlenmeyer flask containing 1 L YPHSM medium [1% (w/v) glucose, 3% (v/v) glycerol, 1% (w/v) yeast extract and 8% (w/v) peptone]. Under these conditions, there was increased protein expression.

Protein extracts were obtained by mechanical procedures from pelleted cells as previously described^36^.

Proteins were purified using *ANTI-FLAG M2* affinity Gel (*Sigma*) packed in 10 mL chromatography columns (Bio-Rad 731–1550). A column with 0.3 mL of affinity gel was equilibrated with TBS (150 mM NaCl, 50 mM Tris-HCl pH 7.4). Elution of the bound FLAG fusion protein was made possible by competition with a solution containing 150 μg/mL FLAG peptide (*Sigma*).

### Protein model analyses

Preselection of the cysteine target residues was done with SSBOND software^37^. Analysis of interfacial surfaces required the Protein Interfaces, Surfaces and Assemblies Service (PISA) at the European Bioinformatics Institute^38^. Analyses of protein structures, target selection for mutagenesis and result analyses involved the software programs PyMol (The PyMOL Molecular Graphics System, Version 1.8 Schrödinger, LLC) and Coot^39^. In most cases, to predict the stability of a mutated structure was used I-Mutant 2.0. software^40^.

### β-PNPG hydrolytic activity measurement and kinetics

Enzymatic activity was measured using *p*-nitrophenyl-β-D-galactopyranoside (β-PNPG). Cellular protein extracts or purified protein preparations were diluted in 150 μL Z buffer (100 mM Na_2_HPO_4_, 40 mM NaH_2_PO_4_, 10 mM KCl, 1.6 mM MgSO_4_). After incubation for 4 min at 30 °C, the reaction was started by adding 150 μL substrate in Z buffer to the enzyme solution. Aliquots (100 μL) of the reaction mixture were stopped at 2 time-points by adding 100 μL 1 M Na_2_CO_3._ Released *p*-nitrophenol was measured by UV absorbance at 400 nm. β-Galactosidase activity is expressed in enzymatic units (U) defined as the amount of enzyme capable of releasing one μmol of product (*p*-nitrophenyl) per min (i.e. μmol min^−1^ mL^−1^) under the experimental conditions.

Kinetic characterization of Kl-*β*-Gal and mutants were based on the assaying of β-galactosidase activity of purified protein samples (as described above) at different substrate concentrations (0–20 mM). Measurements were made in triplicate with 0.3 μg mL^−1^ enzyme. Non-linear fitting was based on least-squares to infer the apparent enzymatic kinetic parameters from Michaelis-Menten plots, using Prism 6.00 for Windows (*GraphPad Software Inc*.).

### Kinetics of lactose hydrolysis

Kinetics of lactose hydrolysis were measured by the glucose produced by the enzyme at different lactose concentrations. Purified samples were diluted in Z buffer. The initial velocity was measured in triplicate with 5.5 μg mL^−1^ enzyme and lactose from 0 to160 mM. The reaction times were 6–20 min at 30 °C. The reaction was stopped by heating to 96 °C for 5 min.

β-galactosidase activity is expressed in enzymatic units (U), defined as the amount of enzyme capable of liberating 1 μmol of product (D-glucose) per min under the experimental conditions (i.e. μmol min^−1^ mL^−1^).

Glucose concentration was measured using the commercial kit D-Glucose GOD-POD (Nzytech). Non-linear fitting by the least squares method was used to infer the apparent enzymatic kinetic parameters from Michaelis-Menten plots (via Prism 6).

### Determination of disulphide contacts

To examine the formation of disulphide bonds in Kl-*β*-Gal and its mutants, mass spectrometry and colorimetric assays were done.

Two different mass spectrometry analyses were performed, Nano-scale LC-MALDI-MS and Nano-LC-QTRAP 5500, as described previously^41^,^42^. Identification of proteins was performed using the Protein Pilot software 4.5 (ABSciex). Search parameters were set with trypsin cleavage specificity, iodoacetamide (IAA) modified cysteine as fixed modifications (when required), biological modification “ID focus” settings, and a protein minimum confidence score of 95% (Detected Protein Threshold >95%, Unused ProtScore >1.3).

DTNB [5,5′-dithiobis (2-nitrobenzoic acid)] colorimetric assay^43^ was also conducted. Protein samples were dissolved in TBS (150 mM NaCl, 50 mM Tris-HCl pH 7.4) with guanidine hydrochloride 3 mM and divided in two aliquots. One of the aliquots was treated with Pierce^TM^ Immobilized TCEP Disulphide Reducing Gel (*ThermoFisher Scientific*) for an hour to reduce disulphide bonds. Samples (non-reduced and reduced) and DTNB solution (2 mM) were mixed in a 1:1 proportion. After incubation at 37 °C for 10 minutes, absorbance of mixtures was measured at 412 nm. DTT was used to make the standard curve. Measurements were made in triplicate. Statistical significant differences (p > 0.05 and p > 0.01) of sulfhydryl groups concentration between non-reduced and reduced conditions of each variant were tested by using a two-tailed Student’s test.

### Thermostability analyses

Thermal stabilities of protein variants (mutants and native enzyme) were determined by 2 procedures. In the first, thermostability was determined by measuring the residual activity of variants after incubation. Protein samples were incubated in Z buffer for different times at 42 and 45 °C.

Differential Scanning Fluorometry (DSF) was used to obtain the melting temperature of both variants^44^,^45^.

The Reaction mix was composed of pure protein (4 μM) and Sypro Orange dye (*Sigma*) at 10x final concentration in a total volume of 25 μL. We used 96-well thin-wall PCR plates (*Thermo*) sealed with *Optical-Quality Sealing Tcape* (BioRad). Samples were incubated for 5 min at 15 °C and heated from 15° to 90 °C with a ramp rate of 0.5 °C min^−1^ in a real-time PCR machine (*iCyclerIQ, BioRad*).

Fluorescence of the dye was continuously monitored. The excitation and emission wavelengths were 490 and 530 nm, respectively. Fluorescence intensity was plotted as a function of temperature and a non-linear Boltzmann fit used the Prism 6 program. The melting temperature was defined as the temperature corresponding to the peak of the first derivative of this curve^45^. Media from 3 independent experiments were obtained.

### Sedimentation velocity assays (SV)

Samples (320 μL) in 50 mM Tris-HCl and 150 mM NaCl at pH 7.4 were loaded into analytical ultracentrifugation cells. Two different enzyme concentrations were used, 0.1 and 0.2 mg/mL. The experiments were carried out at 48 k rpm in a XL-I analytical ultracentrifuge (Beckman-Coulter Inc.) equipped with UV-VIS absorbance and Raleigh interference detection. Sedimentation profiles were recorded at 275 nm. Sedimentation coefficient distributions were calculated by least-squares boundary modelling of sedimentation velocity data using the continuous distribution c(s) Lamm equation model (implemented by SEDFIT 14.7 g)^46^. Experimental *s* values were corrected to standard conditions (water, 20 °C, and infinite dilution) using the program SEDNTERP^47^ to get the corresponding standard *s* values (*s*_20,*w*_).

### Polyacrylamide Gel Electrophoresis (PAGE)

Polyacrylamide Gel Electrophoresis used previously reported procedures^48^.

In the case of the native PAGE, the use of sodium dodecyl sulfate (SDS) in gel and buffers was avoided, and electrophoresis run at 4 °C. In all cases, polyacrylamide gels were stained with *Coomasie Brilliant Blue*^49^.

### Galactooligosaccharide measurement

GOS and lactose concentrations were determined by HPLC (HPLC Waters Breeze I), using a Waters Sugar-Pak column eluted at 90 °C with Milli-Q water at a flow rate of 0.5 mL/min, and a Waters 2414 refractive-index detector.

Reactions involved mixing 0.0006 U (U as defined in lactose kinetics) of pure protein in phosphate buffer 0.1 M (pH 6.8), supplemented with 40% lactose. Samples (500 μL) were incubated at different temperatures and 300 rpm. Samples were taken at 0, 2, 4, 6, 8, 24 and 48 h. Reactions ran at 40 °C.

Carbohydrates were quantified by external calibration, using standard solutions of galactose, glucose, lactose, raffinose and stachyose.

## Additional Information

**How to cite this article:** Rico-Díaz, A. *et al*. Rational mutagenesis by engineering disulphide bonds improves *Kluyveromyces lactis* beta-galactosidase for high-temperature industrial applications. *Sci. Rep.* **7**, 45535; doi: 10.1038/srep45535 (2017).

**Publisher's note:** Springer Nature remains neutral with regard to jurisdictional claims in published maps and institutional affiliations.

## Figures and Tables

**Table 1:** Main properties of Kl-*β*-Gal variants obtained.

Mutant	Subunit Interface involved	Residual activity * change compared with native	Presence of hydrolytic activity (P = presence/NP = not presence)
V2C/H817C	Monomer-Monomer	<10%	P
G37C/S260C	Monomer-monomer	=	P (<20%)
G818C	Monomer-monomer	=	P
R116/T270	Dimer-dimer	>10%	P
H425C/T872C	Dimer-dimer	—	NP
L601C/E922C	Dimer-Dimer	<10%	P
G983C	Dimer-dimer	=	P
R116C/T270C/G818C	Monomer-Monomer Dimer-Dimer	>25%	P

*Residual activity: Remaining activity after 20 minutes incubation at 42.5 °C.

**Table 2:** Peptide fragments with dhA modifications in cysteine sites detected by mass spectrometry analyses.

Mutant variant	Peptide sequence	Aminoacid range	Confidence (%)
R116C/T270C	TFEL**C**SKSIESFEHR	112–126	99
R116C/T270C/G818C	DYKDDDDKS**C**LIPENLRNPK	2–13	99
R116C/T270C/G818C	TFEL**C**SKSIESFEHR	112–126	99
R116C/T270C/G818C	VYDASSLLNEENGNT**C**FSTK	255–274	97.2

Cysteines involved in disulphide bonds formation are marked in bold face.

**Table 3:** Free sulfhydryl concentration of Kl-*β*-Gal and its mutants in non-reduced and reduced conditions determined by the DTNB method.

	Free sulfhydryl groups concentration (μmol/g protein)	
	Non-reduced conditions	Reduced conditions
Kl-*β*-Gal	0.0588 ± 0.0199	0.0593 ± 0.0038
R116C/T270C	0.0897 ± 0,0046	0.1087 ± 0.0014*
R116C/T270C/G818C	0.0862 ± 0.0099	0.1250 ± 0.0054**

The ± sign refers to standard error. *Significance p-value with non-reduce condition <0.05; **significance p-value with non-reduce condition <0.01.

**Table 4:** Kinetic analysis of Kl-*β*-Gal and its mutants.

	β-PNPG			Lactose		
	Km (mM)	Vmax (U/mg)	Vmax/Km	Km (mM)	Vmax (U/mg)	Vmax/Km
Kl-*β*-Gal	2.15 ± 0.52	71.90 ± 4.67	33.44	34.31 ± 8.87	15.99 ± 1.48	0.47
R116C/T270C	2.22 ± 0.25	99.42 ± 3.02	44.78	38.20 ± 10.25	15.24 ± 1.52	0.40
R116C/T270C/G818C	2.47 ± 0.36	146.30 ± 6.11	59.23	34.82 ± 9.49	28.48 ± 2.80	0.82

The ± sign refers to standard error curve fit using the kinetic module of Prism 6.
